# A Study on Yield Criteria Influence on Anisotropic Behavior and Fracture Prediction in Deep Drawing SECC Steel Cylindrical Cups

**DOI:** 10.3390/ma17122872

**Published:** 2024-06-12

**Authors:** Quy-Huy Trieu, The-Thanh Luyen, Duc-Toan Nguyen, Ngoc-Tam Bui

**Affiliations:** 1 Faculty of Mechanical Engineering, University of Economics—Technology for Industries, Hanoi 100000, Vietnam; tqhuy@uneti.edu.vn; 2 Faculty of Mechanical Engineering, Hungyen University of Technology and Education, Hungyen 160000, Vietnam; luyenthethanh@gmail.com; 3 School of Mechanical Engineering, Hanoi University of Science and Technology, 1A-Dai Co Viet Street, Hai Ba Trung District, Hanoi 100000, Vietnam; 4 Innovative Global Program, Shibaura Institute of Technology, Tokyo 135-8548, Japan; tambn@shibaura-it.ac.jp

**Keywords:** anisotropy, yield criteria, deep drawing, SECC steel, fracture prediction

## Abstract

The deep drawing process, a pivotal technique in sheet metal forming, frequently encounters challenges such as anisotropy-induced defects. This study comprehensively investigates the influence of various yield criteria on the anisotropic behavior and fracture prediction in SECC steel cylindrical cups. It integrates Hill’48R, Hill’48S, and von Mises yield criteria in conjunction with Swift’s hardening law to evaluate material behavior under complex stress states. Experimental and numerical simulations assess the anisotropy effects across multiple orientations (0°, 45°, and 90°), revealing intricate relationships between stress criteria and material response. The findings indicate significant discrepancies between isotropic and anisotropic models in predicting fracture heights, emphasizing the importance of selecting appropriate yield criteria. Notably, the von Mises criterion results in lower fracture heights, suggesting higher susceptibility to fractures, while the Hill’48R model aligns closely with experimental data, validated through variations in punch corner radius and blank holder force parameters, with a maximum deviation of 3.23%. Hill’48S displays moderate plastic deformation characteristics.

## 1. Introduction

In contemporary manufacturing, numerical simulations have become indispensable tools for sheet metal forming processes, particularly in industries like automotive production. Stamping processes often encounter challenges such as wrinkles, cracks, and thinning, demanding substantial resources and costs for resolution [1,2,3]. Traditional trial-and-error or experimental-based tool design approaches are progressively being supplanted by finite element simulation systems [4,5,6,7]. Simulation offers a crucial avenue for enhancing product quality, curtailing mold production times, pre-emptively detecting errors before testing [8], and thereby minimizing defective output rates [9].

Finite element methodology, coupled with ABAQUS 6.13 software, is harnessed here to investigate the ramifications of the punch and die shaft deflection on both stamping force and the distribution of wall thickness in SECC sheet billets [10]. In leveraging the Hill model and SECC material’s forming limit curve in numerical simulations, this study predicts two misalignment conditions of punch–mortar interactions—single-axis and multi-axis misalignment. Substantial punch–mortar misalignment is shown to correlate with elevated stamping forces and uneven, thinner cup wall thickness distribution. In previous studies [11,12], numerical simulations were utilized to investigate the effects of various process parameters on the fracture height of cylindrical cups fabricated from SECC. Specifically, parameters such as punch corner radius (Rp), die corner radius (Rd), punch/die clearance (Wc), and blank holder force (BHF) were examined in relation to the forming height of cylindrical cups. The research extends to the optimization of forming height, wherein suitable parameters are determined by evaluating the deviation between simulation and experimental outcomes. Additionally, the study proposes the formulation of a precise mathematical equation to forecast fracture height under diverse machining conditions, with a maximum observed deviation of 4.52% between the mathematical model and simulation. A subsequent investigation [12] analyzed the influence of BHF and limited drawing ratio (Mt) on the formation of cylindrical cups from electrolytically galvanized (SECC) steel billets with a thickness of 0.6 mm. Initial exploratory experiments were conducted to establish the research boundaries of the input parameters. Subsequently, the impact of BHF and Mt on the fracture height of cylindrical cups (H) was assessed. Findings indicate that with a fixed Mt, an increase in BHF from 8 to 12 kN results in a decrease in (H). Conversely, when BHF is fixed and Mt increases from 1.94 to 2.09, the (H) increases. However, as Mt continues to rise to 2.24, the (H) decreases.

Neto et al. [13] have conducted finite element simulations to assess the influence of stress criteria and the methodologies employed to ascertain their parameters. Stress criteria including von Mises [14], Hill’48 [15], and Barlat Yld’91 [16] are examined in the context of the inverted depth drawing of cylindrical cups. Similarly, Dal and his collaborators [17] have scrutinized inner wall wrinkles in the cylindrical cup pressing process, using diverse constitutive models for numerical simulations involving AA5042-H2 aluminum alloy. These models encompass yield criteria such as CPB06ex2, Hill’48, BBC2008-8p, and BBC2008-16p, with both isotropic stiffness and von Mises yield criteria.

In another vein, Said and colleagues [18] have proposed a mathematical model rooted in the plasticity of sheet materials, employing simulation to predict cracks during deep drawing processes. The study introduces a constitutive equation for material anisotropic ductility. Barrera et al. [19] have studied the EK4 material in deep drawing, initially conducting sheet sample pull tests in rolling, diagonal, and transverse directions. The Hollomon–Coulomb hardness models and the Hill48 model were subsequently evaluated and validated through numerical simulations, aligning with Erichsen technology wherein materials experience a primarily biaxial stress state. The predictions obtained demonstrated commendable agreement when compared to corresponding experimental measurements.

Szewczyk et al. [20] conducted friction testing using a specially designed friction simulator and performed uniaxial tensile tests to determine the mechanical properties of the specimens. In their study, they used commonly available oils as lubricants, which are relatively inexpensive compared to the specialized lubricants typically used in sheet metal forming for deep drawing processes. Friction analysis using flat die strip drawing tests was primarily performed on samples with cylindrical or circular surfaces. Djordjević et al. [21] examined a friction model based on the deep drawing process of an AlMg4.5Mn0.7 aluminum alloy sheet sliding between TiN-coated flat contact surfaces under variable drawing forces. Their methodology included analytically predetermined contact pressure functions, allowing for the determination of the relationship between the drawing force and the friction coefficient under various conditions.

The present research employs finite element simulations to meticulously assess and select stress criteria for the accurate prediction of crack heights in cylindrical cups composed of SECC sheets. The central focus lies in scrutinizing diverse stress criteria’s impact on crack height and comparing outcomes with experimental cup height measurements. Specifically, the von Mises, Hill’48R, and Hill’48S stress functions are explored in conjunction with Swift’s hardening law [22], each assessed across orientations of 0°, 45°, and 90° relative to the rolling direction. Anisotropy parameters for the Hill’48 criterion are determined through two distinct approaches. The first involves utilizing anisotropy values r_0_, r_90_, and r_45_ from three uniaxial tensile tests (0°, 45°, and 90° relative to the rolling direction) alongside the uniaxial yield stress σ_0_, as computed via the Hill’48R stress model. The second approach relies on triple stresses σ_0_, σ_45_, and σ_90_, along with the anisotropy coefficient in the tensile direction r_0_, as determined by the Hill’48S model. A comparison between simulation and experimental results underscores the effectiveness of the selected stress criteria, with potential applications for a more precise prediction of fracture heights across a diverse range of cylindrical cups, each characterized by distinct technological parameters.

## 2. Material and Method

The concept of isotropic hardening is fundamental to most analyses of sheet metal forming. Numerous isotropic hardening laws have been developed to accurately characterize the stress–strain behavior of various sheet metals [23]. The Swift model, in particular, is widely applied and effectively represents the stress–strain response of body-centered cubic (BCC) materials, including iron, stainless steel, and high-strength steel. This study focuses on SECC material, an electro-galvanized cold-rolled steel with a thickness of 0.6 mm, conforming to the Japanese standard JIS G 3313 [24]. This steel also corresponds to the ST12 grade under the DIN 1623 standard [25] and is equivalent to the DC01 grade in the EN 10130 standard [26]. The stress–strain relationship utilized in this study is described by the Swift model, expressed as follows:

(1) $$\sigma_{y}=K{\left(\varepsilon_{0}+{\overset{-}{\varepsilon }}^{p}\right)}^{n}\;\mathrm{where}\;\varepsilon_{0}={\left(\frac{\sigma_{0}}{K}\right)}^{1/n}$$

where $\sigma_{y}\;\mathrm{and}\;{\overset{-}{\varepsilon }}^{p}$ represents the yield stress and equivalent strain. The material constants *K*, *n*, and $\sigma_{0}\;$ only are appraised by scrutinizing uniaxial tensile test outcomes on material samples as per the standard procedure.

The determination of material parameters was carried out through conventional uniaxial tensile tests. Experimental stress–strain curves were obtained by measuring the responses along three distinct orientations relative to the rolling direction (RD). The Swift isotropic stiffness model was subsequently developed by fitting the stress–strain curves across all directions, utilizing the coefficients specified in Equation (1). During the numerical simulation process, the material properties of SECC [10] steel were specified as follows: density (ρ = 7.85 × 10^−6^ kg/mm^3^), elastic modulus (E = 184 kN/mm^2^), and Poisson’s ratio (ν = 0.33). The material parameters (K, n, and $\;\sigma_{0}$) were derived from uniaxial tensile tests conducted in the rolling direction, resulting in the following values: K = 473.5 MPa, $\sigma_{0}=152.5$ MPa, and n = 0.226. The anisotropic coefficients, r0, r45, and r90, were determined to be 1.56, 1.21, and 1.8, respectively.

In this study, the anisotropic parameters of the SECC material are assessed using two stress functions: von Mises and Hill’48. The commonly employed isotropic stress function, von Mises stress (Equation (2)), stands as a foundational reference in sheet metal forming analysis.

(2) $$\overset{-}{\sigma }=\sqrt{\frac{1}{2}[{\left(\sigma_{1}-\sigma_{2}\right)}^{2}+{\left(\sigma_{2}-\sigma_{3}\right)}^{2}+\left(\sigma_{3}-\sigma_{1}{)}^{2}\right]}$$

where $\overset{-}{\sigma }$ represents the equivalent stress, while $\sigma_{1},\;\sigma_{2}$, and $\sigma_{3}$ denote the principal stresses along three distinct directions.

For the flat stress case, this is expressed as Equation (3):

(3) $$\overset{-}{\sigma }=\sqrt{\sigma_{11}^{2}+\sigma_{22}^{2}-\sigma_{11}\sigma_{22}+3\sigma_{12}^{2}}$$

For materials characterized by anisotropic properties, the stress–strain curves differ across various directions. The Hill’48 model, acknowledged as the primary anisotropic stress criterion, provides an equivalent stress calculated through Equation (4):

(4) $${\overset{-}{\sigma }}^{2}=H{\left(\sigma_{11}-\sigma_{22}\right)}^{2}+F{\left(\sigma_{22}-\sigma_{33}\right)}^{2}+G{\left(\sigma_{33}-\sigma_{11}\right)}^{2}+2L\sigma_{23}^{2}+2M\sigma_{31}^{2}+2N\sigma_{12}^{2}$$

Here, $\sigma_{11},\sigma_{22},\sigma_{33},\sigma_{11},\sigma_{23}$, and $\sigma_{31}$ signify the stress components in the respective directions, and G, F, H, N, L, and M denote the material constants deduced from experimental data. In the case of plane stress ($\sigma_{33}=0$), Equation (4) is simplified to Equation (5):

(5) $$\overset{-}{\sigma }=\sqrt{H{\left(\sigma_{11}-\sigma_{22}\right)}^{2}+F\sigma_{22}^{2}+G\sigma_{11}^{2}+2N\sigma_{12}^{2}}$$

Two distinct methods are utilized to determine the anisotropic parameters for the Hill’48 model. The first approach employs anisotropy values (r_0_, r_90_, and r_45_) obtained from uniaxial tensile tests (0°, 45°, and 90° relative to RD), combined with uniaxial yield stress (σ_0_) calculated using the Hill’48R stress model. The second method is predicated on triple stress values (σ_0_, σ_45_, and σ_90_) and the anisotropy coefficient (r_0_) calculated using the Hill’48S model. For directions where assessing metal sheet anisotropy proves challenging, parameters defining those properties are assumed isotropic in both Hill’48 approaches, with L and M values set to 1.5.

Alternatively, for the Hill’48 stress criterion operating under flat stress conditions ($\sigma_{33}=\sigma_{23}=\sigma_{31}=0$), the uniaxial yield stress in the θ direction, aligned with the rolling direction, is defined by Equation (6). Consequently, the prediction of uniaxial anisotropy in the same rolling direction θ is facilitated through the utilization of the stress criterion depicted in Equation (7). The process of determining anisotropy parameters for the Hill’48 stress criterion is rooted in the experimental yield stress and the experimental coefficient of the plastic anisotropy of materials. Table 1 provides detailed parameters for the Hill’48R and Hill’48S criteria. Notably, the coefficient specified in Equation (5) corresponds to the Hill’48R model, as outlined in Equations (8a)–(8d), maintaining the same values presented in Table 1.

(6) $$\sigma_{\theta }=\frac{\overset{-}{\sigma }}{\sqrt{\left(F+H\right){sin}^{4}\theta +\left(G+H\right){cos}^{4}\theta +2\left(N-H\right){sin}^{2}\theta {cos}^{2}\theta }}$$

(7) $$r_{\theta }=\frac{H+\left(2N-4H-G-F\right){sin}^{2}\theta {cos}^{2}\theta }{F{sin}^{2}\theta +G{cos}^{2}\theta }$$

The determination of anisotropy parameters based on experimental yield stress and experimental coefficients of plastic anisotropy is presented in Table 1. The approach also involves calculating the Hill’48R and Hill’48S parameters outlined in Table 1.

(8a) $$G=\frac{1}{1+r_{0}}$$

(8b) $$H=\frac{r_{0}}{1+r_{0}}$$

(8c) $$F=\frac{r_{0}}{r_{90}\left(1+r_{0}\right)}$$

(8d) $$N=\frac{\left(r_{0}+r_{90}\right)\left(1+2r_{45}\right)}{2r_{90}\left(1+r_{0}\right)}$$

The coefficients outlined in Equation (5) are consistent with the Hill’48S model, characterized by Equations (9a)–(9d). These specific parameters are extracted directly from the triaxial yield stress values, namely σ_0_, σ_45_, and σ_90_, along with the equi-biaxial tension stress, σ_b_ [27]. Herein, the stress values σ_y_ and σ_0_, are attributed as σ_y_ = σ_0_ = 152.5 MPa and σ_b_ = 165.2 MPa, respectively. The coefficients G, H, F, and N are ascertained according to the indications laid out in Table 1.

(9a) $$G=\frac{1}{2}\left[\frac{1}{{\left(\frac{\sigma_{0}}{\sigma_{y}}\right)}^{2}}-\frac{1}{{\left(\frac{\sigma_{90}}{\sigma_{y}}\right)}^{2}}+\frac{1}{{\left(\frac{\sigma_{b}}{\sigma_{y}}\right)}^{2}}\right]$$

(9b) $$H=\frac{1}{2}\left[\frac{1}{{\left(\frac{\sigma_{0}}{\sigma_{y}}\right)}^{2}}+\frac{1}{{\left(\frac{\sigma_{90}}{\sigma_{y}}\right)}^{2}}-\frac{1}{{\left(\frac{\sigma_{b}}{\sigma_{y}}\right)}^{2}}\right]$$

(9c) $$F=\frac{1}{2}\left[-\frac{1}{{\left(\frac{\sigma_{0}}{\sigma_{y}}\right)}^{2}}+\frac{1}{{\left(\frac{\sigma_{90}}{\sigma_{y}}\right)}^{2}}+\frac{1}{{\left(\frac{\sigma_{b}}{\sigma_{y}}\right)}^{2}}\right]$$

(9d) $$N=\frac{1}{2}\left[\frac{4}{{\left(\frac{\sigma_{45}}{\sigma_{y}}\right)}^{2}}-\frac{1}{{\left(\frac{\sigma_{b}}{\sigma_{y}}\right)}^{2}}\right]$$

In light of the coefficients calculated according to the stress models of Hill’48-R and Hill’48S (Table 1), coefficients *R*_11_, *R*_22_, *R*_33_, *R*_12_, *R*_13_, and *R*_23_ are determined using Equation (10), providing results presented in Table 2.

(10) $$\left\{\begin{array}{c} R_{11}=\sqrt{\frac{1}{G+H}}\\ R_{22}=\sqrt{\frac{1}{F+H}}\\ R_{33}=\sqrt{\frac{1}{F+G}}\\ R_{23}=\sqrt{\frac{3}{2L}},R_{13}=\sqrt{\frac{3}{2M}},R_{12}=\sqrt{\frac{3}{2N}} \end{array}\right.$$

**Figure 1:**
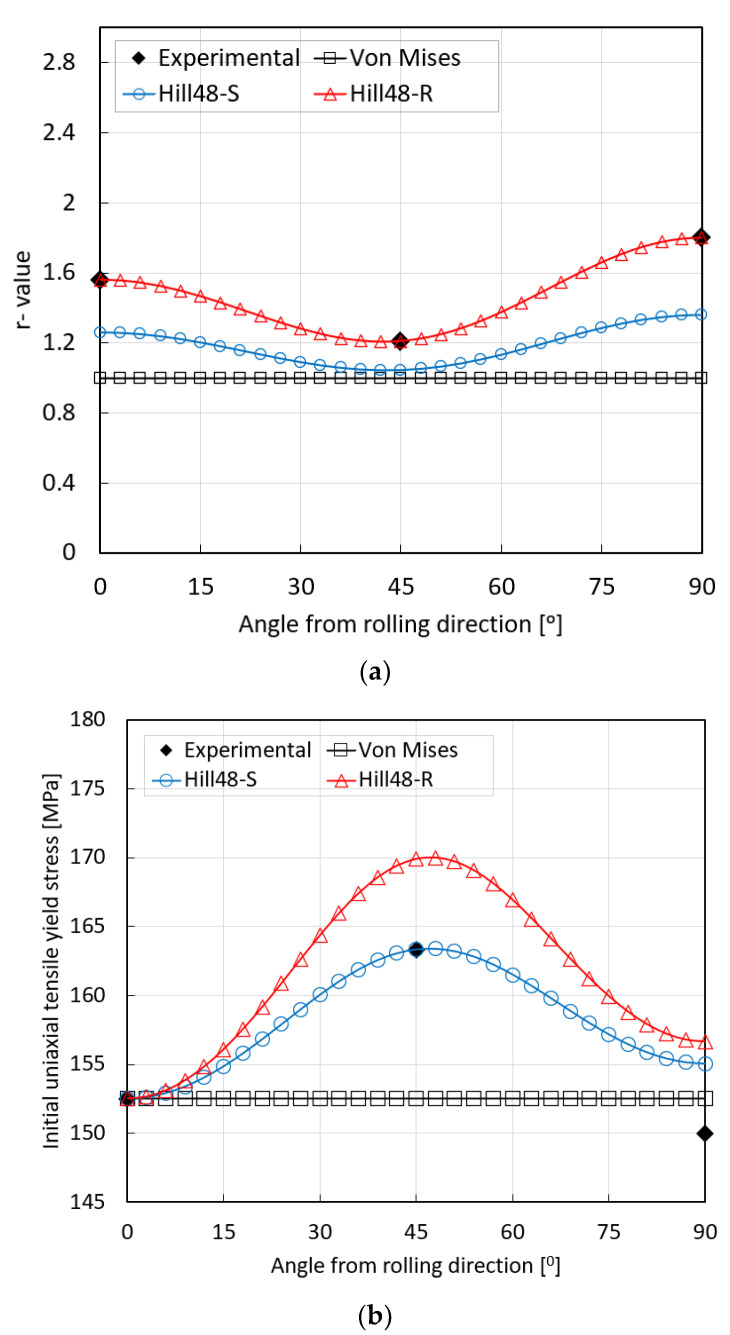
Comparison between experimental and predicted distributions of the anisotropy coefficient (**a**) and initial uniaxial tensile yield stress (**b**) for different stress criteria.

This study critically examines different stress criteria, including von Mises, Hill’48R, and Hill’48S, by comparing them with experimental data. The distribution of uniaxial anisotropy in the plane and the stress distribution in different directions relative to the rolling direction are presented in Figure 1a,b, respectively. Results indicate the Hill’48R criterion aligns closely with experimental values. In contrast, the Hill’48S model exhibits lower dislocation values in the 90° test direction, with a maximum deviation of 0.44. The von Mises model, inherently isotropic, presents a higher deviation of 0.8.

Figure 1b illustrates that all three stress models converge with the experimental value in the 0° direction. However, for the Hill’48R model, in the 90° direction and the 45° direction relative to the rolling direction, the stress is higher than the experimental value. The Hill’48S model aligns with the experiment in the 0° and 45° directions, while in the 90° direction, it predicts a higher value than the experimental one, deviating by 5.04 MPa. Lastly, the von Mises model forecasts a stress value lower than the experimental value in the 45° direction, with a deviation of 10.83 MPa, and in the 90° direction, the stress value is 2.5 MPa higher than the experimental value.

**Figure 2:**
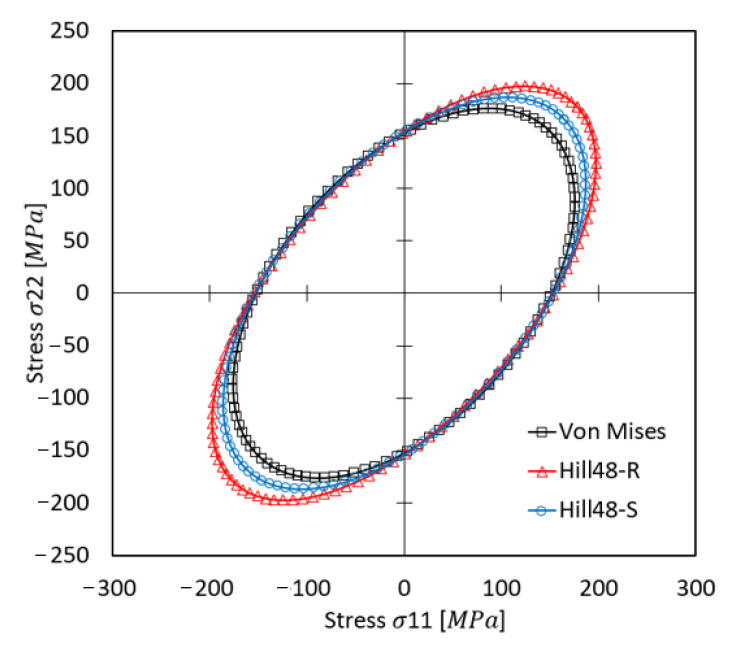
Predicted yield surfaces based on the von Mises, Hill48-R, and Hill48-S criteria.

Figure 2 presents the predicted stress surfaces using different yield criteria for σ_11_ and σ_22_. The von Mises stress model yields a maximum stress value of 176,053 MPa, the Hill’48R criterion stands at 198,088 MPa, and the Hill’48S criterion reaches 186,609 MPa.

In this preliminary analysis, a rigorous assessment is conducted to validate the accuracy of several critical factors in the context of SECC material behavior. These factors encompass the plasticity model, stress model, and anisotropy coefficient. The primary objective is to ascertain their reliability and fidelity in representing the material’s response under various conditions. To commence this evaluation, particular attention is directed towards the distribution of uniaxial anisotropy within the material plane and the corresponding stress distribution. Notably, the obtained data consistently aligns with the experimental data, particularly when considering the rolling direction at 0°. This alignment serves as a significant validation of the models under investigation. Subsequently, the stress–strain curve derived from Swift’s model, specifically applied to the rolling direction at 0°, is leveraged as a pivotal component in simulating the deep drawing process of cylindrical cups. This strategic choice ensures that the simulation closely mimics real-world conditions, capitalizing on the validated model and the associated anisotropy coefficients. In the context of the deep drawing simulation, the forming height is meticulously examined and compared across three von Mises models: the conventional von Mises model, Hill’48R, and Hill’48S. This comprehensive analysis establishes a clear correlation between the chosen models and their ability to replicate the actual deep drawing process. The results of this preliminary analysis, thus far, demonstrate a noteworthy level of consistency between the simulated outcomes and the corresponding experimental benchmarks. This alignment underscores the utility and reliability of the chosen modeling approaches and their capacity to provide valuable insights into the complex behavior of SECC materials during deep drawing processes.

## 3. FE Simulation Model and Experimental Setup for the Deep Drawing Process

### 3.1. Finite Element Model of the Deep Drawing Process

**Figure 3:**
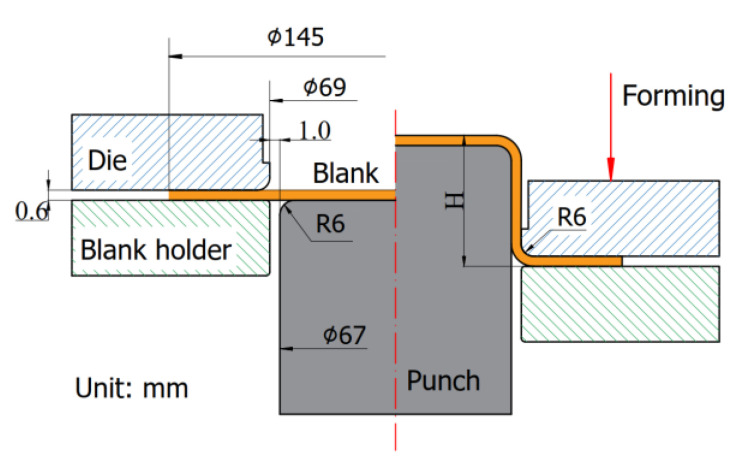
Die set parameters.

This investigation centers on the deep drawing of a cylindrical cup produced from SECC sheet material, which possesses a specific thickness of 0.6 mm. The deep drawing process is characterized by a well-defined configuration, as illustrated in Figure 3. Several critical dimensions play a significant role in defining the geometry and behavior of the produced cylindrical cup. First and foremost, the material thickness (t) of the steel sheet is 0.6 mm, and the cup’s initial diameter (D0) measures 145 mm, contributing to the initial conditions of the forming process. Additionally, the punch’s corner radius (Rp) is set at 6 mm, mirroring the die’s corner radius (Rd) of equal magnitude. These radii values are instrumental in shaping the cup and influencing the material flow during the deep drawing operation. Furthermore, the punch and die clearances, represented by Wc (clearance between the punch and die), are precisely defined at 1 mm. This parameter plays a vital role in governing the deformation characteristics and overall success of the deep drawing process. A paramount focus of this investigation is the determination of the fracture height (H) of the cylindrical cup. The fracture height serves as a crucial parameter for both simulation and experimental analysis. Its accurate assessment involves a meticulous and comprehensive analysis, shedding light on the material’s fracture susceptibility and structural integrity under varying conditions.

**Figure 4:**
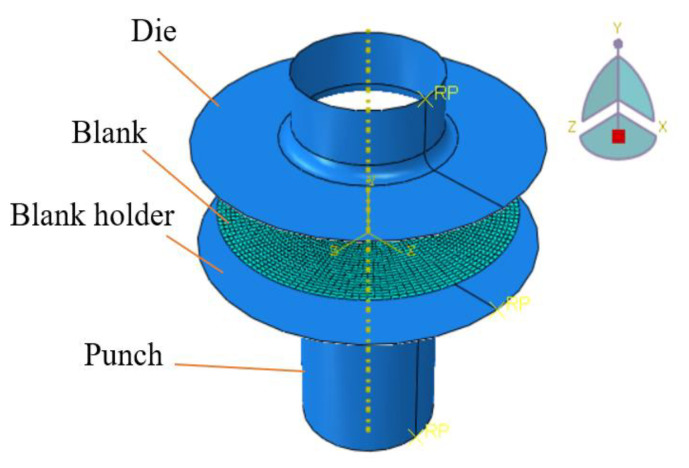
Finite element model of the cylindrical cup’s stamping process.

**Figure 5:**
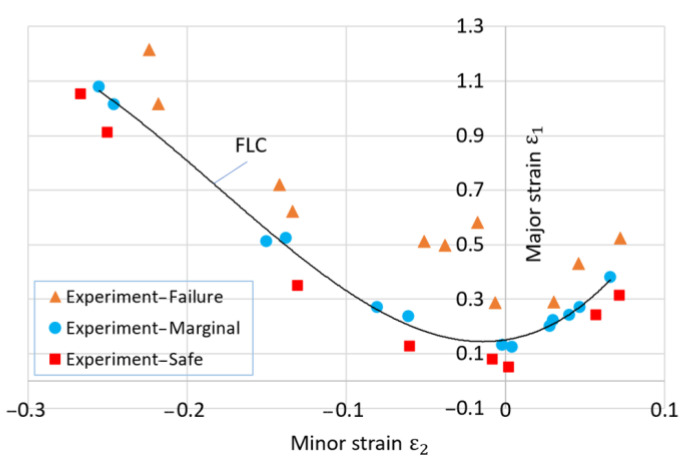
The forming limit curve of the SECC material.

This research utilizes ABAQUS 6.13 software to elucidate the deep drawing process through a 3D finite element model, as illustrated in Figure 4. In the simulation setup, the punch remains stationary while the die and blank execute vertical movements. Within this simulation framework, the mold components—punch, die, and blank holder—are modeled as perfectly rigid parts with shell-shaped cross-sections. Conversely, the blank sheet is modeled as a deformable material. The mesh geometry utilizes S4R elements, which are four-node quadrilateral stress/displacement shell elements with reduced integration and a large-strain formulation. This configuration is meticulously chosen to ensure an accurate representation of the structural behavior. A critical aspect of this simulation pertains to the coefficient of friction, which plays a pivotal role in the interaction between the punch and the workpiece. A coefficient of friction of 0.25 governs this interaction, ensuring realistic and reliable results. Additionally, a uniform coefficient of friction of 0.125 is maintained between the blank holder, punching die, and blank, adhering to established guidelines [28]. In both the simulation and experimental setups, a consistent application of a blank holder force (BHF) at 12 KN is maintained. This parameter serves to standardize the boundary conditions, ensuring meaningful comparisons between the two methodologies. The forming limit curve (FLC) for the SECC sheet material was constructed following the Nakajima test procedure outlined in ISO 12004–2 standards [29]. Figure 5 provides a visual representation of the FLC for this material. Within Figure 5, failure points are denoted by orange triangles, safe points by red and blue square markers, and marginal points by blue circular indicators. FLC is constructed by aligning Marginal points using Matlab R2012b software. Furthermore, Table 1, Table 2 and Table 3 delineate the pivotal parameters influencing the deep drawing process. Of particular importance is the integration of isotropic and anisotropic coefficients linked to the von Mises model, Hill’48R, and Hill’48S models in the numerical simulation of the deep drawing process for the cylindrical cup. This step facilitates a comprehensive exploration of how different yield criteria impact the forming height of the cylindrical cup.

### 3.2. Experimental Methodology for Deep Drawing Process and Measurement of Cylindrical Cup Height

**Figure 6:**
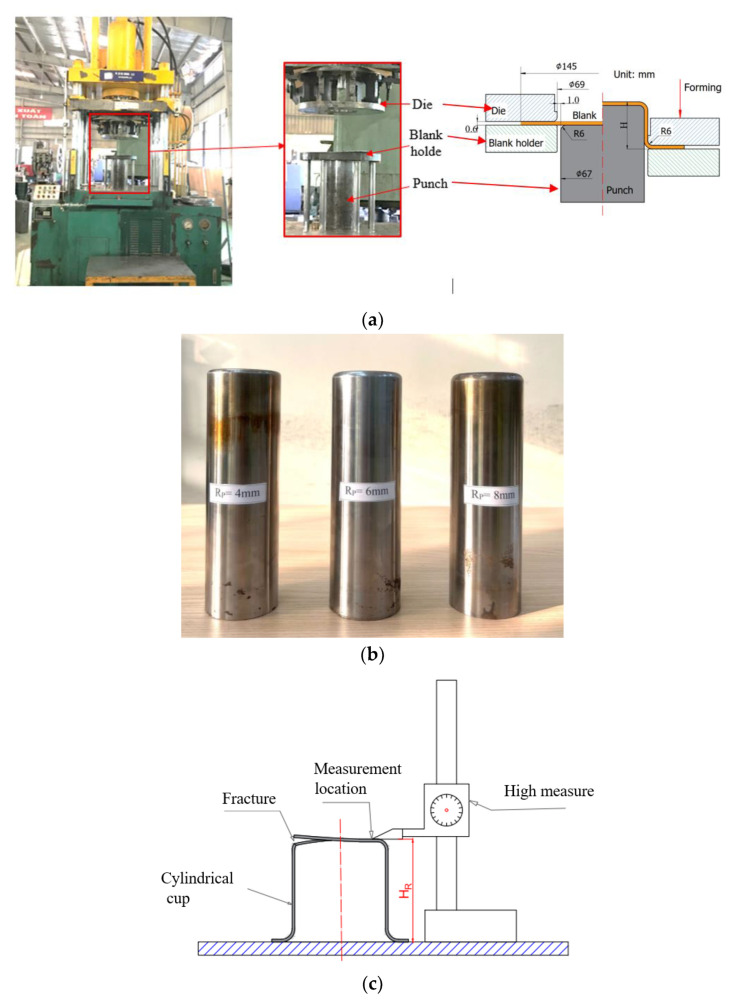
The deep drawing die arrangement (**a**), various punch’s corner radius (**b**), and the height measurement process for a cylindrical cup (**c**).

In tandem with the numerical simulation, experimental verification is conducted through a four-cylinder double hydraulic press model Y28-200. Figure 6a presents the deep drawing die arrangement. Specifically, the die is affixed to the upper hydraulic system, while the punch is secured to the machine table. The movement of the workpiece stop is governed by the hydraulic system positioned at the base of the die machine. Critical components like the punch, blank holder, and die are forged from SKD11 material, having undergone heat treatment for enhanced properties. The mold’s working surface is machined to a surface roughness of Ra = 0.63 μm. Subsequent components of the mold are crafted from S50C material. The process of deep drawing employs specialized oil to diminish friction between the die components and the blank.

For the precise determination of the fracture height (H) of the cylindrical cup following the deep-drawing process, the Japanese Mitutoyo 192–132 height gauge is utilized. As illustrated in Figure 6c, the occurrence of a tear allows for the assessment of the fracture height (H) of the cylindrical cup. The deformed cup is situated on the worktable, aligning the height measurement point opposite the fracture site. The Mitutoyo height gauge provides indispensable features, enabling accurate depth measurements spanning up to 300 mm with a precision of ±0.05 mm.

## 4. Results and Discussion

**Figure 7:**
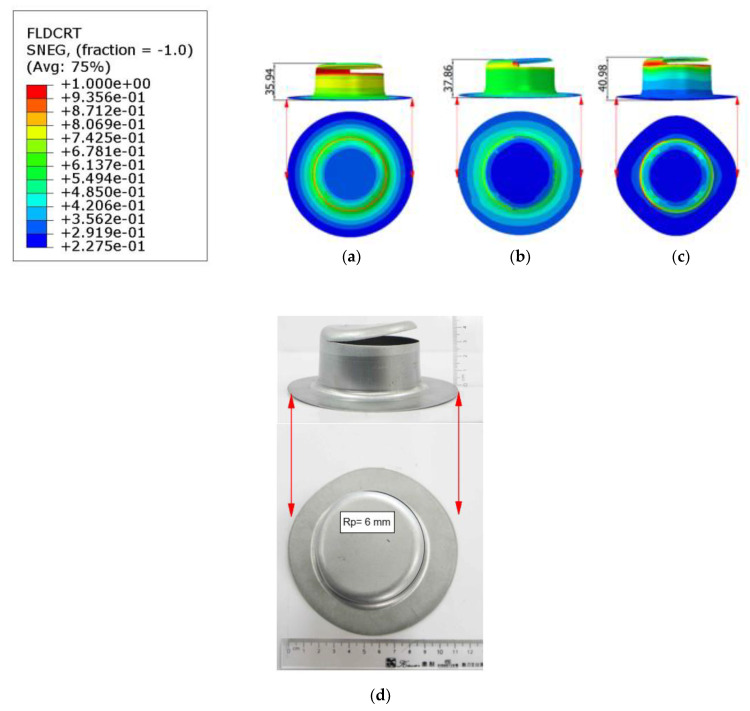
Simulated outcomes using the von Mises stress model (**a**), Hill’48S (**b**), Hill’48R (**c**), and experimental cup (**d**).

The findings of the finite element simulation, which employs Swift’s hardening law and focuses specifically on the 0-degree rolling direction, coupled with the utilization of stress models including von Mises, Hill’48S, and Hill’48R to predict tear height in the context of deep drawing cylindrical cups, have been subject to rigorous scrutiny. Within the simulations, the damage evolution criterion stands as a pivotal element utilized to simulate material failure and fracture. This criterion is defined by the point where the forming limit curve ductile fracture (FLDCRT) value reaches 1.0, indicating a fracture condition to ascertain fracture height (H) in the FEM simulation. To assess the disparities between simulated and experimental tear heights, we have employed the formulation presented in Equation (11). Figure 7a–c have been included to visually represent the simulated outcomes using the von Mises stress model, Hill’48S, and Hill’48R, respectively.

Figure 7a vividly illustrates a uniformly rounded rim profile for the cylindrical cup, suggesting isotropic behavior, in accordance with the predictions of the von Mises stress model. On the other hand, Figure 7b presents a rim profile that initially exhibits a tendency towards an oval shape, hinting at a departure from isotropic behavior. Strikingly, Figure 7c stands out by showcasing a rim profile that closely resembles that of the experimental cup. This observation underscores the remarkable alignment between the simulated outcome and experimental reality. For a quantitative assessment of forming heights and deviations between the cylindrical cups, a detailed comparison between simulation and experimentation is presented in Table 3. Notably, it is observed that the discrepancy between the height predicted by the von Mises stress model and the actual tear height observed in the experiment amounts to 10.42%. Conversely, the deviation between the simulated forming height according to the Hill’48S stress model and the experimental height is notably lower at 5.63%. The smallest discrepancy, only 2.14%, corresponds to the Hill’48R model. This comprehensive comparative analysis serves to emphasize a remarkable alignment between the forming height of the cylindrical cup as predicted by the simulation employing the Hill’48R stress model and the experimental results.

(11) $$\Delta h\left(\%\right)=\left(\left|h_{s}-h_{e}\right|\right)/h_{e}$$

**Figure 8:**
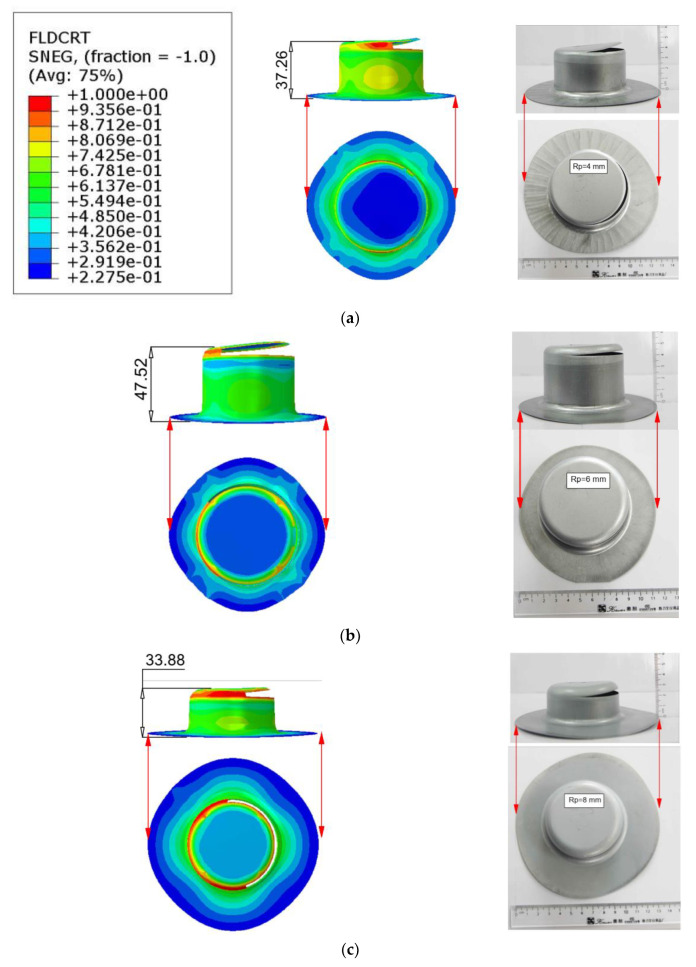

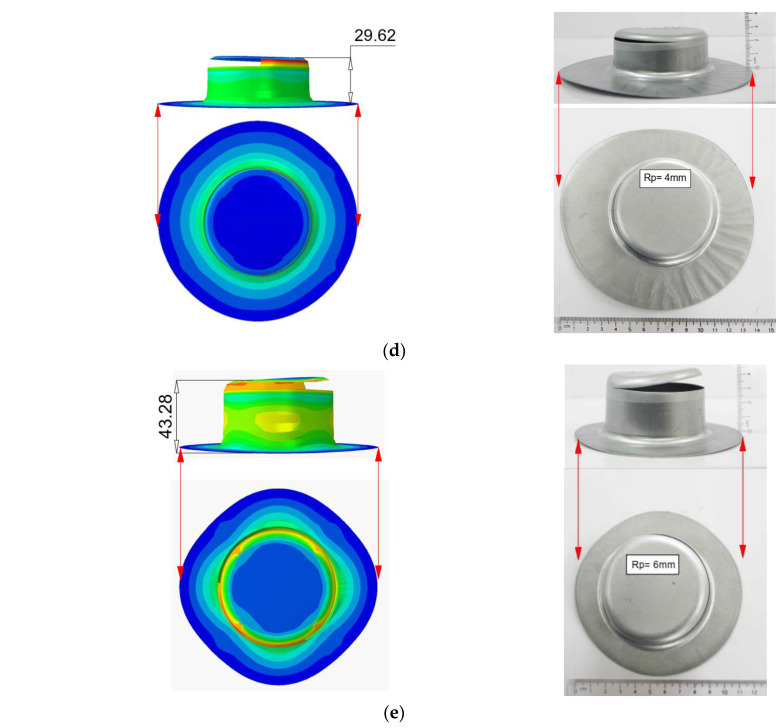
Fracture height of cylindrical cup formation, where simulations and experiments were conducted under varying parameters: (**a**) Case 1 (Rp = 4 mm; BHF = 12 kN), (**b**) Case 2 (Rp = 6 mm; BHF = 10 kN), (**c**) Case 3 (Rp = 8 mm; BHF = 14 kN), (**d**) Case 4 (Rp = 4 mm; BHF = 14 kN), and (**e**) Case 5 (Rp = 6 mm; BHF = 12 kN).

**Figure 9:**
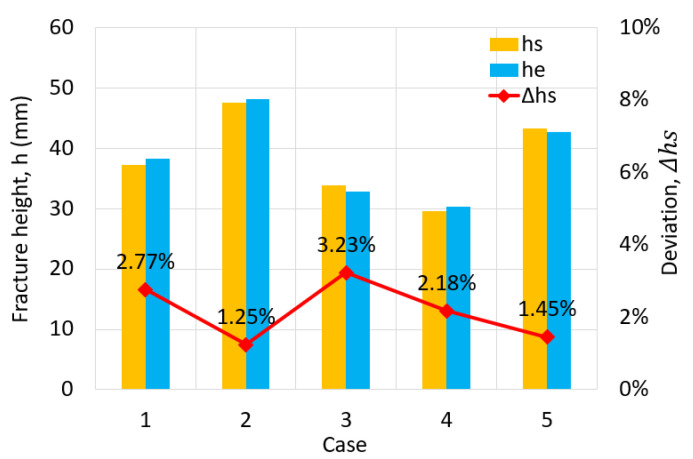
Fracture height and deviation of the cylindrical cup when comparing simulation results with experimental data.

In order to establish the robustness and reliability of the Hill’48R stress model for the numerical simulation of the deep drawing process for cylindrical cups, an extensive verification and confirmation process was conducted. This study aimed to assess the model’s suitability under varying conditions by altering two key parameters: the punch’s corner radius (Rp) and the blank holder force (BHF). It is important to note that all other process parameters remained consistent with previous settings. The deep drawing process underwent thorough simulation and experimentation involving varying punch corner radii and blank holder forces, leading to three distinct scenarios: deep drawing with Rp = 4 mm and BHF = 12 kN, visualized in Figure 8a; deep drawing with Rp = 6 mm and BHF = 10 kN, as portrayed in Figure 8b; and deep drawing with Rp = 8 mm and BHF = 14 kN, depicted in Figure 8c. A meticulous analysis was conducted to scrutinize the outcomes, with a specific emphasis on the resulting forming heights and deviations. These findings have been comprehensively documented in Table 4, providing a quantitative assessment, and further illustrated in Figure 9 to offer a graphical representation for clarity and reference. The results of this investigation reveal a strikingly good agreement between the simulated and experimental fracture heights in all three scenarios. The observed deviations are notably low, further confirming the reliability of the Hill’48R stress model. Specifically, when changing the parameters to Rp = 4 mm and BHF = 12 kN, the deviation is found to be 2.77%. In the case of Rp = 6 mm and BHF = 10 kN, the deviation reduces to just 1.25%, demonstrating a high degree of accuracy. Finally, when altering the parameters to Rp = 8 mm and BHF = 14 kN, the deviation remains modest at 3.23%. Crucially, in each of these scenarios, the simulation successfully replicates the cup’s rim profile, mirroring the corresponding experimental outcomes. This consistency and alignment between simulation and experimentation, even when varying the deep stamping process parameters, underpin the conclusion that the Hill’48R stress model is indeed suitable for subsequent simulations involving SECC sheet materials.

## 5. Conclusions

This study aimed to investigate the correlation between stress models and fracture height in the deep drawing process of cylindrical SECC products. In employing a comprehensive methodology encompassing computation, simulation, and empirical validation, several significant findings have emerged:

- The adoption of Swift’s model-based stress–strain curve, customized to the 0-degree rolling direction and augmented with pertinent anisotropy coefficients, provides a robust framework for simulating cylindrical cup deep drawing. This approach facilitates the precise assessment of forming height, with simulations employing von Mises, Hill’48R, and Hill’48S models demonstrating a close agreement with experimental data.
- The analysis of the simulated rim profile of the cylindrical cup reveals a distinctive oval shape, closely resembling its experimental counterpart. Notably, while the von Mises stress model exhibits a substantial deviation of 10.42% in simulated tear height compared to experimental observations, the Hill’48S model yields a deviation of 5.63%. Conversely, the Hill’48R criterion demonstrates the closest agreement, showcasing a minimal difference of 2.14%. Furthermore, the alignment of the Hill’48R model with experimental values of the r-value and the plasticity of the material is noteworthy.
- The validation of the Hill’48R model’s suitability involved introducing variations in the punch’s corner radius and blank holder force in both simulation and experimentation. Consistently, strong agreement in forming height between simulation and experiment was observed, with deviations ranging from 1.25% to 3.23%. Importantly, the simulation effectively replicates the cup rim profile observed in experimental cups.

In summary, this research elucidates the effectiveness of the Hill’48R stress model in predicting forming height in cylindrical cup deep drawing processes. Moreover, it highlights the significance of considering variables such as the r-value and material plasticity, underscoring the need for further investigation into these aspects in future studies.

## Figures and Tables

**Table 1:** Material Parameters for Hill’48R and Hill’48S.

Label	F	G	H	L	M	N
Hill’48R	0.3385	0.3960	0.6094	1.5	1.5	1.2469
Hill’48S	0.4098	0.4423	0.5577	1.5	1.5	1.3175

**Table 2:** Anisotropy Coefficients for Hill’48R and Hill’48S.

	R_11_	R_22_	R_33_	R_12_	R_13_	R_23_
Hill’48R	1	1.0271	1.1711	1.0968	1	1
Hill’48S	1	1.0167	1.0833	1.0670	1	1

**Table 3:** Comparison of Forming Heights of Cylindrical Cups and Percentage Deviations between Simulation and Experiment.

Fracture height			
Experiment		*h_e_* (mm)	40.12
Simulation	Von Mises	$h_{s-vonMises}$ (mm)	35.94
	Hill’48R	$h_{s-Hill48R}$ (mm)	40.98
	Hill’48S	$h_{s-Hill48S}$ (mm)	37.86
Deviation	Von Mises	$\Delta h_{s-vonMises}$ (%)	10.42%
	Hill’48R	$\Delta h_{s-Hill48R}$ (%)	2.14%
	Hill’48S	$\Delta h_{s-Hill48S}$ (%)	5.63%

**Table 4:** Fracture height and deflection measurements for the cylindrical cup in simulation and experimentation.

Fracture Height			
Case No	Simulation *h_s_* (mm)	Experiment *h_e_* (mm)	Deviation $\Delta h_{s}$
1	37.26	38.32	2.77%
2	47.52	48.12	1.25%
3	33.88	32.82	3.23%
4	29.62	30.28	2.18%
5	43.28	42.66	1.45%

## Data Availability

The original contributions presented in the study are included in the article, further inquiries can be directed to the corresponding author.
